# Costs Of Using “Tiny Targets” to Control *Glossina fuscipes fuscipes*, a Vector of *Gambiense* Sleeping Sickness in Arua District of Uganda

**DOI:** 10.1371/journal.pntd.0003624

**Published:** 2015-03-26

**Authors:** Alexandra P. M. Shaw, Inaki Tirados, Clement T. N. Mangwiro, Johan Esterhuizen, Michael J. Lehane, Stephen J. Torr, Vanja Kovacic

**Affiliations:** 1 Division of Pathway Medicine and Centre for Infectious Diseases, School of Biomedical Sciences, College of Medicine and Veterinary Medicine, The University of Edinburgh, Edinburgh, United Kingdom; 2 AP Consultants, Walworth Business Park, Andover, United Kingdom; 3 Vector Biology Department, Liverpool School of Tropical Medicine, Liverpool, United Kingdom; 4 Bindura University of Science Education, Department of Animal Science, Bindura, Zimbabwe; 5 Warwick Medical School, The University of Warwick, Coventry, United Kingdom; IRD/CIRDES, BURKINA FASO

## Abstract

**Introduction:**

To evaluate the relative effectiveness of tsetse control methods, their costs need to be analysed alongside their impact on tsetse populations. Very little has been published on the costs of methods specifically targeting human African trypanosomiasis

**Methodology/Principal Findings:**

In northern Uganda, a 250 km^2^ field trial was undertaken using small (0.5 X 0.25 m) insecticide-treated targets (“tiny targets”). Detailed cost recording accompanied every phase of the work. Costs were calculated for this operation as if managed by the Ugandan vector control services: removing purely research components of the work and applying local salaries. This calculation assumed that all resources are fully used, with no spare capacity. The full cost of the operation was assessed at USD 85.4 per km^2^, of which USD 55.7 or 65.2% were field costs, made up of three component activities (target deployment: 34.5%, trap monitoring: 10.6% and target maintenance: 20.1%). The remaining USD 29.7 or 34.8% of the costs were for preliminary studies and administration (tsetse surveys: 6.0%, sensitisation of local populations: 18.6% and office support: 10.2%). Targets accounted for only 12.9% of the total cost, other important cost components were labour (24.1%) and transport (34.6%).

**Discussion:**

Comparison with the updated cost of historical HAT vector control projects and recent estimates indicates that this work represents a major reduction in cost levels. This is attributed not just to the low unit cost of tiny targets but also to the organisation of delivery, using local labour with bicycles or motorcycles. Sensitivity analyses were undertaken, investigating key prices and assumptions. It is believed that these costs are generalizable to other HAT foci, although in more remote areas, with denser vegetation and fewer people, costs would increase, as would be the case for other tsetse control techniques.

## Author Summary

Sleeping sickness remains a serious threat in Sub-Saharan Africa. The disease is normally controlled by medical screening of the human population and treatment of individuals found to be infected. The disease is transmitted by tsetse flies but vector control is rarely used for control. A major reason given is that is too expensive in resource poor settings. We have developed a novel technology based on insecticide treated screens (= tiny targets) to control flies more cost-effectively. A 250 km^2^ field trial of tiny targets has been performed in Northern Uganda and we made use of this to undertake a full costing analysis of tiny target technology. The cost of the operation was costed at USD 85.4 per km^2^. This represents a major reduction in the cost of tsetse control. The reductions are largely due to the low costs of tiny targets and to the ease with which they can be deployed.

## Introduction

Tsetse control technologies and their mode of delivery are evolving all the time. A major purpose of this evolution is to develop approaches that can reduce the incidence of human and animal African trypanosomiasis (HAT and AAT) more cheaply and/or more effectively. Measuring cost-effectiveness accurately, and in such a way that different operations and approaches are fully costed and can be validly compared, is essential to underpin decision-making in this field [1]. This paper analyses the costs of an actual field operation using the new technology of tiny targets undertaken in Arua District, Uganda in 2012/2013 whose ultimate aim was to reduce transmission of HAT by controlling *Glossina fuscipes fuscipes* [2].

In contrast to analyses of tsetse control operations primarily undertaken to control AAT, costs of such operations undertaken in HAT foci have only been intermittently reported on in the entomological literature [3]. With the development of lower cost devices a major concern, these reports have focussed on their unit cost and related this cost to the km^2^ and the human population ‘protected’. The term ‘protected’ was introduced to indicate the area and the people within that area who benefited from tsetse control, as against the much more restricted area of tsetse habitat where traps or targets were actually deployed. Thus, excluding manpower, the newly developed Vavoua trap was reported as costing about half as much as the standard biconical and pyramidal traps [4]. Four projects using traps and screens in HAT foci published cost-effectiveness estimates for Côte d’Ivoire [5], Congo [6], Equatorial Guinea [7] and Uganda [8]. Coincidently these all relate to the 5-year period 1986–1990. In Uganda, the project area’s population was around 320,000; the other three all worked in HAT foci containing about 25,000 people. All cite trap costs, which can be compared to levels today by converting from local currencies to United States dollars (USD) at the historical rates applicable at the time, and then updated to current (2014) prices by applying the USD inflation rate (http://inflationdata.com/inflation/Inflation_Rate/HistoricalInflation.aspx historical data) which for this period yields a factor ranging from 2.16 to 1.88, thus roughly doubling all prices. Thus, at 2014 prices in Côte d’Ivoire screens cost USD 6.6 and Vavoua traps USD 13.6; in Equatorial Guinea [7] pyramidal traps costing an estimated USD 9 [9] were used. Meanwhile, in the Congo [6], pyramidal traps costs USD19 and villagers were supplied with a repair kit for the traps costing USD 6, giving an average annual cost per trap of USD10, all at 2014 prices. In Uganda [8] pyramidal traps costing USD 6 were being used on a large scale, and the newly developed mono-screen trap [10] cost USD 8.8 at 2014 prices. In 2012/13 pyramidal traps were bought for use in Uganda at a cost of USD 10—indicating that their relative price has remained surprisingly stable over time.

In order to evaluate the relative cost-effectiveness of traps and targets/screens, these monetary costs needed to be assessed alongside measures of effectiveness against tsetse populations. For traps, catches can be compared (e.g. [10]). However, in order to compare traps and screens a more sophisticated metric is required because targets do not retain the flies killed. The tsetse control operation analysed here follows on from a decade’s research into increasing the ‘cost-effectiveness’ of the targets themselves, measured in terms of tsetse caught or killed per m^2^ of cloth for *G*. *f*. *fuscipes* [2,11] and *G*. *palpalis palpalis* [12]. This standard metric has made it possible to compare the effectiveness of the classic targets or biconical / pyramidal traps, in use since the 1980s as described above, with much smaller devices. The amount of fabric required gives a clear and measurable indicator of trap/target cost which can be compared over time and across countries and currencies. For *G*. *p*. *palpalis*, the killing efficiency of a medium-sized horizontal target design 0.5 m^2^ was shown to be 6 times greater than of the classic 1 m^2^ target [11]. The adoption of the ‘tiny’ 0.125m^2^ (0.5 X 0.25 m) target for use in this trial follows directly from these studies [11] showing the killing efficiency of *G*. *f*. *fuscipes* per m^2^ to be between 5.5 and 15 higher than for 1 m targets and up to 8.6–37.5 greater than for biconical traps. Similar results were recently obtained by [13] for *G*. *p*. *palpalis*, showing 0.25 m^2^ targets to be promising as cost-effective devices, but using relative catches as a metric. The cost-effectiveness of different traps for *G*. *f*. *fuscipes* has also been studied using catch per linear m of fabric as a metric [14].

The four historic projects cited above went on to calculate trap costs per person protected, which at 2014, prices came out to USD 11, USD 11, USD 0.5 and USD 1 for Côte d’Ivoire, Equatorial Guinea, Congo and Uganda respectively. For Côte d’Ivoire, adding the deployment costs for fuel and vehicle maintenance plus trap replacement and reimpregnation with insecticide increased the cost to USD 13 per person protected. Although labour and staff costs were not costed [5] provided a detailed inventory of all inputs, including people’s time alongside full instructions for estimating the costs of operations. In Uganda, adding cost for staff, labour and transport increased the cost per person protected to USD 2. Costs were also given per km^2^. These costs reflect very different population densities in the HAT foci from 17 per km^2^ in Côte d’Ivoire to 100 in Uganda. Devices were also placed at very different densities with 25 per km^2^ protected in Côte d’Ivoire, 10 to 15 per km^2^ in Uganda. Costs per km^2^ in Uganda, at 2014 prices, worked out at USD 85, rising to USD 179 if staff, labour and transport were included; in Côte d’Ivoire the cost per km^2^ protected was higher at USD 217. These costs refer to the first year of deployment. For all the projects, it was thought that costs would fall in the second year of operation, with trap life sometimes extending beyond one year and deployment sites having been selected and prepared.

However, a full analysis of the cost-effectiveness also has to include all delivery costs. It has long been known that the tsetse control techniques described as “expensive” and” high-tech” and usually deployed on a larger scale, such as aerial spraying and the sterile male technique, used on their own, require less expensive ground level support and thus have apparently lower delivery costs than targets and traps, since flying time is usually included in the ‘core’ cost of the technology. As far back as the late 1970s it could be shown that the differential between the apparently high cost of helicopter spraying and ground spraying was greatly eroded when the full delivery costs for ground spraying were included [15] and the same was true for targets and aerial spraying [16]. More recent comparisons [1] also indicate that while total costs of bait technologies (whether stationary: traps/targets or live: insecticide-treated cattle) can be substantially lower, in relative terms, their delivery costs are substantially higher in relation to their core costs (insecticides and traps/targets) than is the case for than aerial spraying or the sterile male technique (core costs of insecticide, sterile males and flying time). The need to reduce delivery costs for the bait technologies was part of the reason why many projects have tried to involve local communities, not just by informing them about the objectives and benefits of using traps and targets, but also in terms of contributing labour and ensuring traps /targets remained in place and in working order [5]. However, community involvement has had mixed success [17], with the needs of communities often being treated as secondary to the entomological objectives. Although not part of a project budget, inputs by community members impose an economic cost on that community, so that an economic analysis should value these inputs.

Lastly, whereas the costs of traps and targets or insecticide can be reduced, delivery costs do not necessarily decline proportionately. For this reason, simply multiplying the trap or insecticide cost by a constant to estimate the delivery cost is unlikely to be reliable. To date, apart from the meticulous detailed information recorded by and the preliminary estimates made for the use of insecticide-treated cattle in southeastern Uganda and reported in [1] there is no published accurate assessment of the delivery costs of such an operation in a HAT focus. Accordingly, alongside the entomological monitoring of the control operation in Arua reported by [18] an important component of the project’s work was the detailed recording and pricing of all inputs.

## Materials and Methods

**Fig 1:**
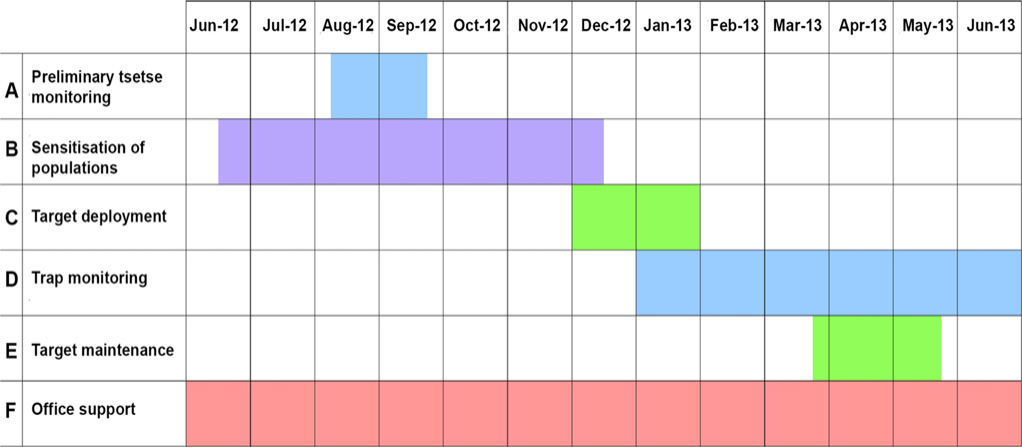
Timing of project activities from June 2012 to June 2013.

The study focussed on a control operation using tiny targets which covered 250 km^2^. Work began in June 2012 with a sensitisation operation, and continued to the end of June 2013, thus covering a period of 13 months. It was split into six sub-activities, spread over that period as given in Table 1 and illustrated in Fig. 1. To monitor costs for each activity, a data sheet was kept, recording the number of days spent in the field (Table 1), staff deployment, labour hired, vehicles used and kilometres travelled, use of other capital items such as global positioning sets (GPS), laptop computers, specialist items (traps, targets and extension materials) and all other running costs (e.g., fuel and oil, vehicle maintenance, hired transport, stationery, GPS batteries, protective clothing for staff, assorted minor consumables). In this way all field and non-field costs were recorded and both variable and fixed costs were fully accounted for. Office overheads were also monitored. The information thus collected provides a full set of data for an actual field operation.

**Table 1:** Component activities of tsetse control work and dates undertaken.

Activity	Dates	Duration	Field days
A. Preliminary tsetse monitoring using traps	06/08/12–23/09/12	48	20
B. Sensitisation of local populations	11/06/12–13/12/12	185	100
C. Target deployment	04/12/12–01/02/13	59	31
D. Trap monitoring	01/01/13–28/06/13	138	101
E. Target maintenance	21/03/13–19/05/13	99	33
F. Office support	01/06/12–30/06/13	394	0
**Entire operation**	**01/06/12–30/06/13**	**394 (449)**	**(285)**

**Note**: Figures in brackets are linear totals of all days spent. Activities overlap, so these exceed the 394 day time period.

**Fig 2:**
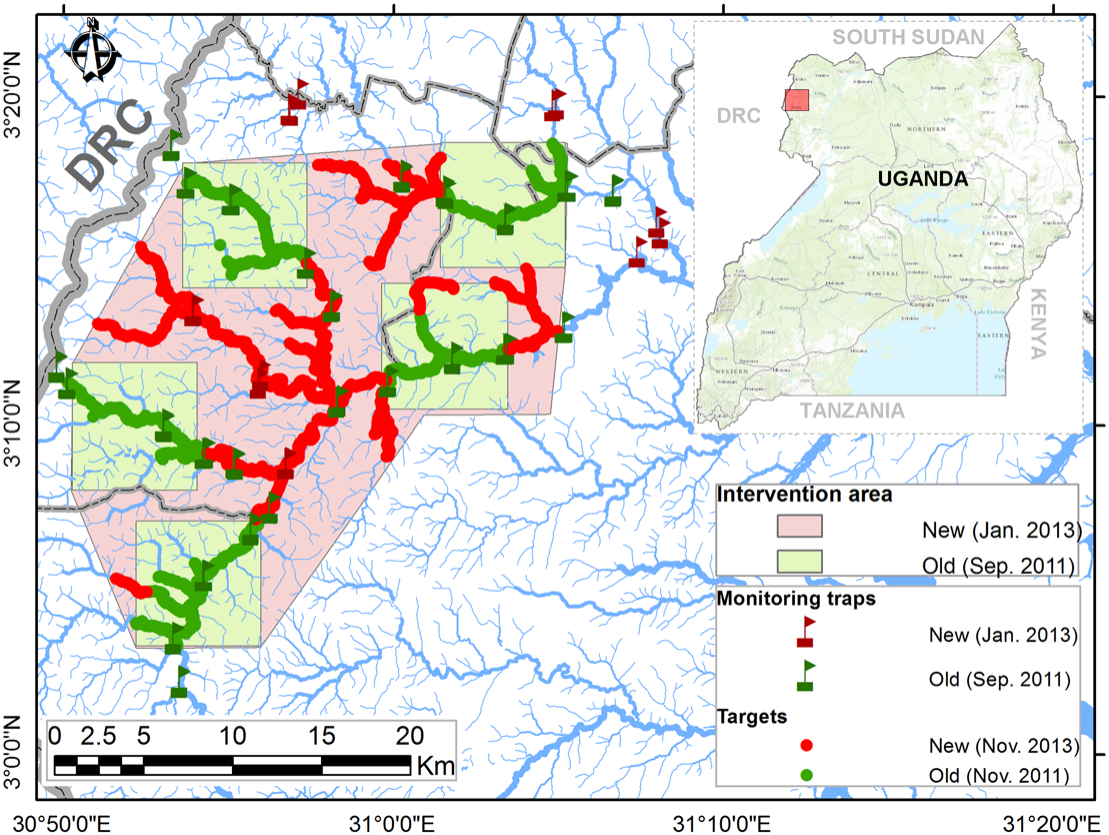
The intervention area.

The operational area on which these costs are based consisted of the area surrounding five blocks, each of 7 x 7 km, which were the subject of a control operation initiated in 2011 (Fig. 2). At the beginning of December 2012, the control area was enlarged from ~250 km^2^ (5 x ~50 km^2^) to 500km^2^. The work done in the ‘new’ areas was carefully logged and separated from that done in the five original ‘old’ blocks (Fig. 2). These areas are contiguous and there was little additional travel between locations. Accordingly, all costs were divided by 250 to produce a cost per km^2^ controlled.

The costing methodology adopted was the ‘full costing’ approach described in [1]. By clearly itemising cost components, the calculations undertaken here are presented so as to enable effective comparison with those presented for other operations.

The overall objective was to produce a replicable costing at current prices for a field tsetse control operation run by local staff in the Ugandan context. Thus costs were adjusted so as to remove the purely research components. The organisation and supervision of the work was undertaken by an academic research team composed of an anthropologist and three entomologists. Supervisory staff inputs were costed at the salaries and travel allowances paid to a Ugandan senior entomologist, for the time spent in the field and on administrative duties. This included a proportionate allowance for weekends and holidays. Each district in Uganda has an entomologist responsible for vector control. Two categories of preparatory work were costed: sensitising local populations and a preliminary tsetse survey. Two costings for the survey are presented, one as actually incurred, the other for a streamlined operation with no research component. The sensitisation programme was treated as a ‘capital’ investment—which would be valid for at least three years before follow up activities were needed.

Within each component activity, the ‘full costs’ for this operation were calculated as follows. Depreciation was estimated for all capital items which outlasted the 13-month project duration. The relevant items and the assumptions used are listed in Table 2. For non-durable items (fuel and oil for vehicles, labour, travel allowances and/or per diems for staff, protective clothing for staff, backpacks, slashers, pangas, stationery, GPS batteries, extension materials and refreshments for villagers during village meetings) the actual recorded expenditure was used. Although the life of traps can exceed one year, they were classified along with targets as ‘specialised equipment’ and their working life was conservatively estimated at 150 days deployment.

**Table 2:** Depreciation assumptions for capital items used by the project.

	Unit cost USD	Years usable	Annual depreciation USD
4x4 Toyota pickup truck	27,000	3	6,500
Office motorbike (new)	5,116	4	1,279
Trap attendant’s motorbike	1,262	3	421
Bicycle	92	2	46
Laptop (average cost)	1,051	3	350
Printer	85	3	28
Generator for office	1,500	5	300
Office furniture (total cost)	837	4	209
GPS unit	548	4	137

**Note**: The Toyota pickup was bought second hand and at end of 3 years the potential resale value was estimated to be between $5,000 and $12,000. No resale value was assumed for the other items. Note that figures are converted from other currencies and rounded. Items costing under USD 50 were not itemised separately, but are included in the activity cost summaries as appropriate. Four high specification laptops were purchased, the cost given here is an average. GPS units were shared across activities and also used for the ‘old’ target area. The estimated share of depreciation was based on 100 days use per annum.

However, for a project undertaken within an existing government structure, some capital and recurrent costs would be spread over several activities, of which tsetse control in a specific area would only be one. In the case of this project, as explained above, in addition to the parallel control work undertaken in the ‘old’ project’s five 50 km^2^ squares, a substantial proportion of the time was allocated to research. Thus only 25% of the office overheads were allocated to the project being costed. Also, for this reason only a share of basic salaries, reflecting the time spent on the project, was included for entomologists. Similarly, for some items (e.g., motorbikes, GPS sets, laptops, traps) an appropriate share of their annual use (based on kilometres travelled or days used, as recorded in the data sheets) was attributed to the project. However, for the 4x4 pickup truck, the cost was based on the total recorded distance travelled which was virtually all for this 250 km^2^ control project.

Prices were all converted to US dollars (USD) mainly from 2012/13 Uganda Shillings (UGX) (and occasionally other currencies). The average rate for the project period was 2615 UGX = 1 USD, ranging from 2416 to 2700 (http://www.oanda.com/currency/historical-rates/). The rate of 2615 UGX = 1 USD was applied throughout. Other currencies used were the British pound (GBP) and the Euro (EUR), whose conversion rates to 1 USD for the period were 0.6382 GBP and 0.7755 EUR. Total costs are rounded to the nearest USD without removing the effects of rounding, thus in some cases the totals will appear not to add up, but all individual figures are accurately rounded. Costs per km^2^ are rounded to the nearest USD 0.1. All costs are at market prices applicable at the time and place where they were incurred and include the cost of shipping to Uganda as relevant.

## Results

### Preliminary tsetse monitoring using traps

Before undertaking tsetse control in the new enlarged area, a preliminary entomological survey was undertaken. The objective of this was to identify suitable sites for locating traps. Traps were deployed and visited daily for 3 days, 1 to deploy and 2 to monitor. The total number of field days was 20, deploying 8 traps (Table 3). The work was undertaken by a senior entomologist and driver, using the 4x4 pickup truck. Total travel was just over 2000 km, accounting for 40% of the vehicle’s mileage during the study period. Some use was also made of the office motorbike. Pangas and slashers were used to cut down the vegetation around the traps sites. The costs are summarised in Table 3. Total costs came to USD 5901, which works out at USD 23.60 per km^2^. The costs were dominated by cost of depreciation (44%) and maintenance (25%) of the pickup truck, reflecting its low annual travel of some 5000 km and relatively high maintenance costs, which were only to some extent offset by its low depreciation, both reflecting the fact that it was 15 years old. Staff salaries and allowances accounted for a further 23% of costs, and fuel 6%.

**Table 3:** Activity A: Preliminary monitoring—as actually undertaken.

Item and category	USD
**Depreciation on Capital Items**	
4x4 pickup truck (40% of USD 6,500)	2600
Office motorbike (2.5% of USD 1,279)	32
GPS units (2 units, USD 127 each, 20% share)	55
**Subtotal depreciation**	**2697**
**Recurrent expenditure**	
***Specialized equipment***	
Traps for monitoring (8, USD 13 each, used for 20 days, cost share)	14
***Vehicle running costs***	
4 x 4 pickup truck fuel (2,041 km)	317
4 x 4 pickup truck share of annual overheads (40% of USD 3,721)	1,488
Office motorbike fuel (120 km)	10
Office motorbike share of annual overheads (2.5% of USD 344)	9
***Staff***	
Team leader—principal entomologist (PE) grade	652
Team leader field allowance	115
Driver	602
***Other***	
Stationery (pens and notebook)	2
GPS batteries (4)	4
Slashers and pangas (share of cost)	1
**Subtotal recurrent costs**	**3,214**
**Overall total**	**5,901**
**Cost per km^2^**	**23.60**

During the course of the project, the reliance on the pick-up truck was gradually reduced, and teams of trap and target attendants were trained. They accessed the project area either by motorbike or bicycle, sometimes using public transport, transporting traps and targets in backpacks. To investigate the impact of this technological and logistical improvement, the preliminary monitoring costs were recalculated (Table 4) based on the timings achieved in activity D (monitoring the actual target deployment) and allowing for more intensive monitoring (12 traps and 25 field days, monitoring a total of 100 trap sites, or 4 per 10 x 10 km square). This intensity is equivalent to the monitoring undertaken in study zone in 2010 when work first began in Arua area. The work would be done by a team of two trap attendants using a motorbike. This approach would allow for considerable cost savings, reducing the total cost to USD 1281, or USD 5.12 per km^2^.

**Table 4:** Activity A: Preliminary monitoring—if trap attendants carried out the work.

Item and category	USD
**Depreciation on Capital Items**	
Office motorbike (10% of USD 1,279)	128
Trap attendants’ motorbike (50% of USD 421)	210
GPS unit (25% share of USD 127)	34
**Subtotal depreciation**	**373**
**Recurrent expenditure**	
***Specialized equipment***	
Traps for monitoring (12, USD 10 each, used for 25 days, cost share)	20
***Vehicle running costs***	
Office motorbike fuel (450 km)	69
Office motorbike share of annual overheads (2.5% of USD 344)	34
Attendants’ motorbike fuel and oil (3500 km)	227
Attendants’ motorbike maintenance	42
***Staff***	
Team leader—principal entomologist (PE) grade	198
Team leader field allowance	17
Trap attendants	287
***Other***	
Stationery (pens and notebook)	8
GPS batteries (4)	5
Slashers and pangas	1
**Subtotal recurrent costs**	**908**
**Overall total**	**1281**
**Cost per km^2^**	**5.12**

### Sensitisation of local populations

Before undertaking the sensitisation campaign preparatory research activities were [19] undertaken. This highlighted people’s wariness, and in some cases fear, of the targets and traps, and underlined the need for an effective public awareness initiative. The campaign proceeded in several steps. Three different sensitization materials were developed in English and translated into Lugbara: letters for communities, information leaflets and flip-charts used for training and for house-to-house sensitisation.

Preliminary meetings with sub-county authorities were carried out and they were briefed about the project activities and sensitization campaigns planned in their area. All the villages in the new control area were identified and mapped, using geographical positioning systems (GPS). This work was initiated by the research team, led by an anthropologist, and then taken over by the target attendants. In total 130 villages were involved in the campaign. The costs (Table 5) are based on what was actually experienced, although the speed of work varied, with the initial research team of 4 managing to walk 12 km a day and identify 18 villages a day (4.5 per person per day). When this was taken over by the target attendants, whose task included this work and then later placing and renewing targets, the rate fell to 1.2 villages per person per day. Eight training meetings were organised for the village health teams (VHTs) at the sub-county headquarters at which refreshments were provided and travel costs paid. VHTs, consisting of two volunteers chosen by each village, are part of the health delivery system in Uganda. VHTs were paid two daily lunch allowances, which corresponded to the usual national VHT rates. Out of the 260 VHT’s, 257 came for training and then returned to their villages to carry out two days of house-to-house sensitization using picture-based flip charts and samples of targets. They were requested to place information sheets on the usual village notice boards and trees. VHTs were moving on foot or using their own bicycles, so no extra transport cost was included in this exercise. On the third day the research team recollected sensitisation materials and the sensitization forms on which the number of people who received the message in house-to-house campaign was recorded. On this occasion VHT members received their lunch allowances and transport fees. This was followed by training meeting for VHTs from another sub-county, where flip charts and targets were reused. In total 8,713 households were visited and 56,983 people received the message.

**Table 5:** Activity B: Sensitisation.

Item and category	USD
**Depreciation on Capital Items**	
4x4 pickup truck (40% of USD 6,500)	2,600
Office motorbike (5% of USD 1,279)	64
Bicycles (4 bicycles, USD 46 each, 50% share)	92
GPS unit (4 units, USD 127 each, 50% share)	274
**Subtotal depreciation**	**3,030**
**Recurrent Costs**	
***Specialized equipment***	
Letters to the community (150, USD 0.41 each) information leaflets (300, USD 0.76 each) and house to house sensitisation forms (360, USD 0.04 each) plus transport from Kampala (USD 19)	324
Flip charts (160, USD 3.82 each, used for 2 campaigns, 50% share)	306
***Vehicle running costs***	
4 x 4 pickup fuel and oil (2014 km)	339
4 x 4 pickup share of annual overheads (40% of USD 3721)	1488
Office motorbike fuel, repairs and oil (245 km)	22
Office motorbike share of annual overheads (5% of USD 344)	17
Target attendants’ travel costs	444
***Staff***	
Team leader and other entomologist both at principal entomologist (PE) grade	2336
Entomologists’ field allowances	126
Driver	701
Translators	325
Target attendants (107 field days)	614
Village health teams (VHTs) payments for travel to and attendance at meetings and lunch allowances during house-to-house sensitisation	1461
***Other***	
Stationery (pens, notebooks, printer cartridges, folders, photocopying and paper)	58
GPS batteries (49)	84
Refreshments for VHTs	173
T-shirts with logo for sub-country chiefs (8)	46
**Subtotal recurrent costs**	**8,864**
**Overall total**	**11,894**
**Cost attributed to this operation, if spread over 3 years**	**3,965**
**Cost per km^2^**	**15.86**

The campaign took place over six months and its costs are summarised in Table 5. The total cost came to USD 11,984. Transport accounted for 43% of costs, labour and staff a further 47%, with only 5% required for the extension materials. It is considered that at least three years would elapse before further sensitisation activities would be needed. Accordingly a third of the costs were allocated to the one-year tsetse control operation, coming to USD 15.86 per km^2^.

In addition, during the course of the project, trap and target attendants undertook sensitization regularly on an informal basis as part of their daily routines: talking to people washing by the rivers, or working in fields. This had no cost implication, as the full cost of their work is included in the activities described below.

### Target deployment

The target deployment activity began in early December 2012, and has been described in detail [18]. Targets were deployed at about 6 per km^2^ when averaged over the whole area, but at a higher density in the riverine habitat as shown in Fig. 2). Because they are so close to each other, the targets in the ‘new’ project area costed here show up as a green dotted line, whereas those in the ‘old’ project area form a red dotted [18]. A total of 1,536 were deployed, and 1,551 provided for in the cost estimate, thus allowing for a slight excess. Targets were manufactured by Vestergaard-Frandsen (Lausanne Switzerland) and shipped from Vietnam. The cost per target was USD 1. Effective target life was assumed to be more than six months and less than a year (see activity E) so targets were treated as recurrent rather than capital cost items. The cost of shipping and insurance varied greatly. In storage tiny targets have a long shelf life—estimated to be about two years. A rapid air consignment cost USD 0.40 per target, lower cost air transport was quoted at USD 0.17 for 10,000 targets and USD 0.126 for 50,000. Larger quantities could be sent by sea, at an estimated cost of USD 0.045 for 100,000 and USD 0.012 for 500,000. If larger scale target deployment were coordinated by the Ugandan government, the sea route would be preferable. In these cost calculations, a cost of USD 0.10 was used, on the assumption that targets would mostly be transported by sea. In order to deploy the cloth targets, wooden supports had to be prepared and glued, a task under taken by the ‘target fixer’.

The deployment was done by teams with bicycles. Targets and supports were transported in backpacks. For the more distant sites, the target teams used public transport (‘boda-boda’ taxi motorbikes and small pick-up trucks) to reach the deployment zone and were paid transport allowances to fund this. The total cost for the activity came to USD 7,370, or USD 29.5 per km^2^ (Table 6). The single largest cost item was the targets, accounting for 27.5% of costs, followed by labour at 25%.

**Table 6:** Activity C: Target deployment.

Item and category	USD
**Depreciation on capital items**	
4x4 pickup truck (8% of USD 6,500)	520
Office motorbike (1.5% of USD 1,279)	19
Bicycles (8 bicycles, USD 46 each, 10% share)	37
GPS unit (6 units, USD 127 each, 33% share)	271
**Subtotal depreciation**	**847**
**Recurrent costs**	
***Specialized equipment***	
Targets (1551, USD 1.1 each)	1,706
Sticks for targets (4833, UGX 150 or USD 0.0574 each)	277
Glue and rubber ties	36
***Vehicle running costs***	
4 x 4 pickup fuel (360 km)	55
4 x 4 pickup share of annual overheads (8% of USD 3721)	298
Office motorbike fuel, repairs and oil (57 km)	9
Office motorbike share of annual overheads (1.5% of USD 344)	5
Rent of vehicle for 3 days	172
Target attendants (10 for 31 field days) transport allowances	1182
***Staff***	
Team leader—principal entomologist (PE) grade	593
Team leader field allowance	23
Target fixer (for 1551 targets, UGX 150 or USD 0.0574 each)	89
Target attendants (10 for 31 field days)	1778
***Other***	
Stationery (marker pens and notebooks)	17
GPS batteries (109)	131
Slashers and pangas (9)	16
Gumboots and overalls (2 sets)	38
Backpacks (8)	92
MTN Airtime	6
**Total recurrent costs**	**6,523**
**Overall total**	**7,370**
**Cost per km^2^**	**29.48**

### Trap monitoring

Once a substantial proportion of the targets were deployed (end of December, 2012) the monitoring activity began (Fig. 1). This continued until the end of the evaluation period. Traps were deployed in the new control zone at 12 sites every twice a month for 3 days, and monitored, as illustrated in Fig. 2. Some of the new trap sites were outside the project area, in order to monitor the impact of the control operation on the boundaries of the treated area. This work was done by the trap attendants, who were supplied with motorbikes. The payment modalities gradually evolved—from allowances to reclaiming actual costs. In the cost calculations (Table 7) all the costs were based on the fuel required for the actual mileage and the associated maintenance costs plus depreciation. The total cost for this activity came to USD 2,250 which worked out at USD 9.00 per km^2^. Vehicle running and depreciation accounted for 46% of this cost, and labour a further 29%.

**Table 7:** Activity D: Trap monitoring.

Item and category	USD
**Depreciation on Capital Items**	
Office motorbike (4% of USD 1,279)	64
Trap attendants’ motorbikes (3 motorbikes, 33% of USD 421)	416
**Subtotal depreciation**	**480**
***Specialized equipment***	
Traps for monitoring (12, USD 10 each, 25% use, half of 6 month period)	60
***Vehicle running costs***	
Office motorbike fuel (241 km)	21
Office motorbike share of annual overheads (5% of USD 344)	17
Attendants’ motorbike fuel and oil (7,447 km)	438
Attendants’ motorbike maintenance	84
***Staff***	
Team leader—principal entomologist (PE) grade	428
Team leader field allowance	34
Trap attendants	660
***Other***	
Stationery (pens and notebooks)	11
Grease	15
Slashers and pangas	2
**Subtotal recurrent costs**	**1,770**
**Overall total**	**2,250**
**Cost per km2**	**9.00**

### Target maintenance

The target maintenance operation began at the end of March 2013. All target sites were visited and targets were repaired or replaced as required, the vegetation around them cleared, etc. By the end of the evaluation period 950 targets had been replaced. The modalities were the same as for the initial deployment, with trap attendants using bicycles or hiring local transport to access the intervention areas. However, the time required was much less, since the sites for the targets had already been determined and only some vegetation clearance was required. The costs are summarised in Table 8. The total costs came to USD 4,290 or USD 17.16 per km^2^. The main cost item was vehicle running (34%) followed by targets (29%) and labour (24%).

**Table 8:** Activity E: Target maintenance.

Item and category	USD
**Depreciation on Capital Items**	
Office motorbike (1% of USD 1,279)	13
Bicycles (7 bicycles, USD 46 each, 10% share)	32
GPS unit (6 units, USD 127 each, 30% share)	247
**Subtotal depreciation**	**292**
**Recurrent costs**	
***Specialized equipment***	
Targets (950, USD 1.1 each)	1,045
Sticks for targets	166
Glue and rubber ties	38
***Vehicle running costs***	
Office motorbike fuel, repairs and oil (50 km)	12
Office motorbike share of annual overheads (1% of USD 344)	3
Rent of vehicle for 2 days	115
Target attendants’ transport allowances (including extra for more distant sites and occasional motorbike taxi)	1308
***Staff***	
Team leader—principal entomologist (PE) grade	99
Team leader field allowance	17
Target fixer (for 950 targets, UGX 150 or USD 0.0574 each)	54
Target attendants (10 for 31 field days)	981
***Other***	
Stationery (pens and notebooks)	11
GPS and AA batteries	74
Slashers and pangas	3
Gumboots and overalls (3 sets)	57
Backpacks (2)	15
**Total recurrent costs**	**3,999**
**Overall total**	**4,290**
**Cost per km^2^**	**17.16**

### Office support costs

Lastly, it is important in field-based projects such as this not to neglect the costs of administering and organising the work. The costs of each field activity include non-field days (itemised in Table 1), mainly for supervisory and research staff. Over and above this it was necessary to run an office, allowing internet access, other communications and processing of data for research purposes as well as routine administration and organisation. The cost components are itemised in Table 9. The total cost of running the office for the evaluation period came to USD 8,724. The office served the two 250 km^2^ control operations. About half the time was taken up with research. Accordingly, 25% of the total cost was attributed to the ‘new area’ control operation costed in this paper. This worked out at USD 8.72 per km^2^.

**Table 9:** Activity F: Office support costs.

Item and category	USD
**Depreciation on Capital Items**	
4x4 pickup truck (5% of USD 6,500)	325
Office motorbike (2% of USD 1,279)	26
Laptops (4 laptops, each 50% of USD 350)	701
Generator	300
Office furniture	209
Office equipment	69
**Subtotal depreciation**	**1,630**
**Adjusted for 13 month period**	**1,765**
**Recurrent Costs (13 months)**	
***Vehicle running costs***	
4 x 4 pickup fuel and oil (250 km)	39
4 x 4 pickup share of annual overheads (5% of USD 3721)	186
Office motorbike fuel, repairs and oil (100 km)	7
Office motorbike share of annual overheads (2% of USD 344)	7
***Staff***	
Cleaning	348
Guards	1,655
***Other***	
Generator running and servicing	301
Rent	2600
Electricity and water	1034
Stationery and consumables for office uses	239
Mobile phone top-ups	522
**Sub-total recurrent costs**	**6,938**
**Overall total**	**8,724**
**Cost share (25%) attributed to this operation**	**2,181**
**Cost per km^2^**	**8.72**

**Note**: Office furniture: table, chairs, curtains, shelves. Office equipment: fan, mobile phone, electricity stabiliser, extension cable and printer. Four researchers worked on the project using high specification laptops. For a control operation usually one, or at most two, laptops would be used, so a 50% share was included.

### Overall cost

Combining the costs from the six component activities produced the results given in Table 10. The overall total for the control work in the ‘new area’ was USD 21,337, coming out at USD 85.4 per km^2^ or USD 13.8 per target deployed. Of this over half (55%) was for deploying and maintaining targets. The cost of the targets themselves came to 13% of total project costs. By expenditure category, the single largest cost component was transport (35%) followed by labour (24%).

**Table 10:** Cost summary for all activities.

	A	B	C	D	E	F	
	Prelimin-arysurvey	Sensitis-ation	Target deploy-ment	Trap monitor-ing	Target mainten-ance	Office support	Totalcost
** Percentage breakdown within activities**							
**Depreciation on capital items (%)**							
Vehicles	26.4	23.2	7.8	21.3	1.0	4.4	11.5
Other equipment	2.7	2.3	3.7	0.0	5.8	15.9	4.6
**Total capital**	**29.1**	**25.5**	**11.5**	**21.3**	**6.8**	**20.2**	**16.1**
**Recurrent expenditure (%)**							
Specialised equipment	1.6	5.3	27.4	2.7	29.1	0.0	16.7
Vehicle running	29.1	19.4	23.4	24.9	33.5	2.7	23.1
Staff Salaries	15.4	25.5	8.0	19.0	2.3	0.0	10.9
Staff field allowances	1.3	1.1	0.3	1.5	0.4	0.0	0.6
Labour	22.4	20.2	25.3	29.3	24.1	23.0	24.1
Consumables and other	1.1	3.0	4.1	1.2	3.7	54.1	8.4
**Total recurrent**	**70.9**	**74.5**	**88.5**	**78.7**	**93.2**	**79.8**	**83.9**
** Summary of total costs by activity**							
**USD total (250 km^2^)**	**1,281**	**3,965**	**7,370**	**2,250**	**4,290**	**2,181**	**21,337**
**USD per km** ^**2**^	**5.1**	**15.9**	**29.5**	**9.0**	**17.2**	**8.7**	**85.4**
**Share of total cost (%)**	**6.0**	**18.6**	**34.5**	**10.6**	**20.1**	**10.2**	**100.0**

Note: 33% of the cost of sensitisation and 25% of the cost of office support is allocated to total cost, as explained in Sections Band F. Specialised equipment consists of traps (A,D), targets (C,E) and printed extension materials (B).

### Sensitivity analysis

In order to test the robustness of the cost estimates, a range of sensitivity analyses was undertaken (Table 11). These looked first at the impact on overall costs of cost increases of a third in crucial components: targets, traps, labour, senior staff and fuel and public transport costs. The most sensitive items were labour (8.0% increase) and fuel and public transport costs (6.1%), reflecting their relative share in total costs. Secondly, the implications of varying some of the key assumptions made in the cost estimation were examined. This showed that while varying the share of office overheads allocated to the tsetse control operation had only a limited impact, if the sensitisation programme had to be repeated every two rather than every three years, costs would increase by 9.3%. Using the preliminary survey as an example showed that if just this single activity were undertaken using senior staff and a vehicle rather than local staff with bicycles and motorbikes, the overall cost would increase by over 20%.

**Table 11:** Sensitivity analysis.

Hypothetical change	Impact on total cost per km^2^(%)
**Cost increases (by 33%)**	
Price of targets	4.3
Traps	0.1
Labour	8.0
Senior staff (entomologist, anthropologist)	3.5
Fuel, oil and payments for public transport	6.1
**Time lapse between sensitisation campaigns**	
Reduced to 2 years	9.3
Increased to 4 years	-4.6
**Share of office overheads**	
Reduced to 20%	-2.0
Increased to 33%	3.4
**Preliminary survey**	
Area covered increased by 50%	6.4
Using senior staff and a 4 x 4 vehicle	21.6

## Discussion

It is important to set these results in context. As stated in [18], a fully inclusive cost of USD 85.4 per km^2^ involves a marked reduction on earlier estimates. If office overheads, sensitisation and preliminary studies (which account for 35% of costs), are excluded the figure comes out as a ‘field cost’ of USD 55.7 per km^2^, which is the figure that should be compared to the costs published in much of the literature. Referring back to Fig. 2 and the methods section, it should be noted that cost are given for the whole area ‘protected’ rather than per mile of riverine habitat treated. In historic terms this cost is well below the USD 179 (at 2014 prices) noted by [8] also for Uganda with 10–15 traps per km^2^. In terms of contemporary estimates it is also far lower than the USD 556 estimated by [1] for 10 traps per km^2^. The human population density in the control zone was estimated at 500 per km^2^, based on [20] together with gridded data obtained from http://www.afripop.org/. Thus the cost per person ‘protected’ is thus very low, at USD 0.17.

The results of the sensitivity analysis (Table 11) help to explain why the operation was so-cost effective, and to underpin a discussion of the factors which might limit this cost-effectiveness. Looking first at the cost of targets, one of the reasons this type of operation is much less expensive than those undertaken in the late 1980s, as reported on above, is that targets now come made of fabric which has been pre-impregnated with insecticide, so repeat impregnations are not required. Also, when comparing to operations targeting other *Glossina* species, especially those of the *morsitans* group, it should be noted odours are not required as bait here. However, while the target cost was fixed at USD 1 by the supplier, the costs of shipping from Vietnam, where the targets were made, fluctuated a lot. A simple 33% increase in the cost of targets would take the overall cost of the operation up by 4.3%. As explained above, based on the quotes received and in the hope that shipments could be by seas, the cost used in this study was USD 0.10 per target, a figure which seems a reasonable compromise. If the transport cost were doubled to USD 0.20 per target, the cost of the project would rise to USD 86.5 per km^2^. Shipping by sea is only feasible for large quantities, so, in order to keep costs low an in-country project would need to be implemented in several sites of the type studied and to buy in bulk. The only other specialist cost items—traps and extension materials—account for 0.1% and 1.0% of total costs, so cost increases in these items would have little overall impact on the project.

Looking at the other items for which price increases were costed in Table 11, the most significant is labour. Labour accounts for 24.1% of all costs, so increasing it by a third would result in an 8.0% increase in project costs. The high reliance on labour, whether government employees, locally sourced or provided by the community is a characteristic of the bait technologies. In this project in particular, the low overall cost was achieved by first training target/trap attendants who did much of the work and second by providing them with motorcycles, bicycles or hired local transport, thus reducing dependence on large project-owned vehicles. This was only possible because the small targets can be easily transported in a backpack, so that one person can carry up to 30 tiny targets. The impact of this is nicely demonstrated by comparing the two costings for preliminary surveys (Tables 3 and 4). The cost reduction from USD 23.6 to USD 5.1 per km^2^ is achieved by limiting senior staff involvement to supervision and by replacing the use of a 4x4 project vehicle with a good quality office motorbike and cheaper motorbikes used by the target attendants. The more expensive option would involve 21.6% higher costs (Table 11). Another unknown in relation to the preliminary survey is how extensive an area needs to be covered. In this project, some knowledge of the basic area already existed. If a new project was targeting a completely unknown area, or a region where previous tsetse surveys were out of date, the preliminary survey may need to be more extensive. A 50% increase in the area surveyed would increase overall costs by 6.4% (Table 11) and larger increases would have a linear impact on cost increases, if done at the same intensity. However, this survey was intended to help site targets, and it could be argued that a survey aimed simply at identifying future intervention sites might cover a larger area, but at a lower trapping intensity.

Ensuring that local populations want, support and understand the tsetse control measures is key to success [5,8,17]. Pre-intervention attitudes and knowledge in local populations highlighted that effective sensitisation would be a vital part of this project [21]. One unknown in the costings was, for a longer control operation, after how long and to what extent it would be necessary to visit communities again and reinforce the information provided at the start of the operation. As explained above, the village representatives continued to remind their communities of the purpose of the control activities, as did the trap and target attendants in the course of their work. For the latter, easy contact with local people was facilitated by their mode of travel using bicycles or motorcycles. Accordingly a compromise figure of three years was decided on—so that the sensitisation activity was effectively ‘depreciated’ over a 3 year period. If it were deemed necessary to repeat the operation in full after two years, the cost of the operation would increase by 9%. On the other hand, if nothing more had to be done for four years, the cost of the operation would be reduced by 4.6% (Table 11), which is a more likely scenario.

Attributing a share of the office overheads, for what was in many respects more a research than a control operation was also somewhat subjective. Sensitivity analysis indicated that varying the 25% proportion initially allocated from 20% to 33% had only a small impact on total costs (Table 11). Again, as was the case for the preliminary survey assumptions, the differences between the full cost of the office overheads, and those attributable to the control operation illustrates how costs are reduced if the research components are removed.

Thus, looking at individual components of the costs which might change in value or whose underlying assumptions might need changing, it is clear that foreseeable changes in the cost or quantities of a single item are unlikely to have a major effect on the costs. Of course, if several changes occur together, then a cumulative effect would be more significant. But overall, the costs can be described as robust. However, it is important to state that these costs apply to a specific tsetse control operation, and thus they only include what was done as part of that operation. Wider studies—such as surveys of trypanosomiasis in human and livestock populations were not undertaken as part of this tsetse control operation, although livestock were sampled as part of the separate research activities. The area was well known as a focus of HAT and controlling the disease in livestock was not a driver for the work. There were no separate training courses. The entomologists were already qualified in tsetse control. The target and trap attendants received their training in GPS use, trap placement and monitoring and target placement and repairs in the field, under supervision from the entomologists. On the sensitisation side, project staff were similarly trained by the anthropologist. The existence of the VHT’s within the Ugandan health service meant that at the village level, a support network for this type of work already existed. Long term monitoring will also provide more detail on target life and replacement rates. There may be some cost reductions in a second deployment as deployment costs in year two could fall because sites have already been identified and trap and target attendants are implementing well practiced routines.

Lastly, it is important to ask—how applicable will these costings be to similar work undertaken in other parts of Africa? Differences are likely to be experienced at three levels. The first one involves different prices, salary structures and a different organisational set up at the level of government services. Secondly, there are also important issues around economies of scale and shared resources to consider. For example, these costs assume the presence of a district entomologist who could allocate a costed share of his time to a particular vector control activity; shipping costs for targets are very dependent on scale, etc. These costings apply to a relatively small area—but should be regarded as ‘lean’, in the sense that almost all resources are fully used and no spare capacity is included. If even smaller areas were targeted, some extra travel from area to area might be required. Also, by spreading the cost of sensitisation over three years, the programme is implicitly assumed to go on for that long. Both of these levels are country and project specific. The third level is ecological, integrating a number of factors. Although the maintenance of *T*. *b*. *gambiense* HAT foci relies on the presence of a human reservoir, population densities can vary greatly: from under 20 per km^2^ as discussed above for the forest zone of Côte d’Ivoire [5] and the 500 per km^2^ estimated for the north-western Uganda study zone costed here. In the *T*. *b*. *rhodesiense* focus of south-eastern Uganda [8], where cattle have been shown to be the major reservoir [22] of the disease, the human population density at the time was 100 per km^2^. In areas with lower human population densities, tsetse habitat is likely to be more dense, access more difficult, terrain could be rugged, overnight stays or camping may be required and local labour be less easy to recruit. All of these factors will drive cost per km^2^ upwards and in areas of low human population density the cost per person protected will rise steeply. Ultimately, in some isolated or rugged areas it might not be possible to rely on all of the lower cost forms of transport, but the benefit of using cheaper and more portable targets will be maintained.

All the considerations discussed above (sensitivity to changes in price and assumptions, price and organisational differences, ability to harness economies of scale, accessibility and its links to human and livestock population density and tsetse habitat) would apply equally to any ground-based tsetse control technology, and to some extent to all vector control methods. Thus while the actual cost levels achieved in this exercise may not be replicable in every situation, the principles on which the cost savings are based will be: low cost delivery using motorbikes or bicycles and local labour together with a cheap and highly portable target with a high killing efficiency.
